# Clinical outcomes following balloon aortic valvuloplasty

**DOI:** 10.1136/openhrt-2020-001330

**Published:** 2020-09-08

**Authors:** Anda Bularga, Rong Bing, Anoop SV Shah, Philip D Adamson, Miles Behan, David E Newby, Andrew Flapan, Neal Uren, Nick Cruden

**Affiliations:** 1 Centre for Cardiovascular Science, University of Edinburgh Division of Health Sciences, Edinburgh, UK; 2 Edinburgh Heart Centre, Royal Infirmary of Edinburgh, Edinburgh, UK; 3 Christchurch Heart Institute, University of Otago, Dunedin, New Zealand

**Keywords:** aortic valve disease, valvuloplasty, percutaneous valve therapy

## Abstract

**Background:**

Balloon aortic valvuloplasty (BAV) remains a treatment option for the selected patients with severe aortic stenosis. We examined clinical outcomes and predictors of prognosis in patients undergoing BAV for severe aortic stenosis.

**Methods:**

We identified all patients undergoing BAV from January 2010 to March 2018 (n=167) at a single transcatheter aortic valve implantation (TAVI) centre. Patient demographics, investigations, subsequent interventions and clinical outcomes were obtained from electronic health records.

**Results:**

Patients undergoing BAV were elderly (median age 80, IQR 73–86 years) and half (n=87, 52%) were male. All-cause mortality at 30 days and 12 months was 11% and 43%, respectively. Reduce ejection fraction (EF 30%–50%: HR 1.76, 95% CI 1.05 to 2.94; EF <30%: HR 1.90, 95% CI 1.12 to 3.20) was the only independent predictor at baseline of overall mortality. Median survival was 212 (IQR 54–490) days from the index procedure. Mortality at 1 year was lowest in patients who subsequently underwent TAVI or SAVR but high among those who had no further interventions or those who had a repeat BAV (14%, 19%, 60%, 89% respectively, log-rank p<0.001).

**Conclusion:**

BAV as a bridge to definitive aortic valve intervention in carefully selected patients offers acceptable outcomes. These contemporary observational findings demonstrate the ongoing potential utility of BAV in the TAVI era.

**Key questions**

**What is already known about this subject?**

- Improvement in procedural techniques, equipment and patient assessment together with the introduction of transcatheter aortic valve implantation (TAVI) has led to a revival in the use of balloon aortic valvuloplasty (BAV) in the management of symptomatic severe aortic stenosis. The contemporary role of BAV includes symptom palliation, test for symptom improvement and bridge for definitive therapy through percutaneous or surgical aortic valve replacement.

**What does this study add?**

- BAV can be performed with a low procedural risk of death or stroke, it has a role in symptom improvement and when used as a bridge to definitive aortic valve intervention in carefully selected patients can offer favourable outcomes in the current TAVI era. However, long-term outcomes in patients selected for an isolated BAV remain guarded. Furthermore, repeat BAV is associated with high mortality.

**How might this impact on clinical practice?**

- BAV remains a useful interventional tool in the management of symptomatic severe aortic stenosis patients with for short-term symptomatic relief or as a temporising measure to allow further evaluation and consideration of definitive management. Selection of patients for BAV is an important consideration and has to be done on an individual patient basis taking into account clinical, functional and echocardiographic markers.

## Introduction

Aortic stenosis is now the most common valvular heart disease in the developed world and its prevalence is rapidly rising due to an ageing population.^1–4^ The progression of calcific valve degeneration is associated with adverse prognosis, even in asymptomatic patients.^1–4^ To date, the only definitive therapy remains aortic valve replacement.^5^ The current treatment paradigm for aortic stenosis has been established over many years, with major society guidelines^6–8^ recommending intervention for patients with severe aortic stenosis who are symptomatic or have reduced left ventricular ejection fraction (LVEF), with weaker recommendations in asymptomatic patients with early evidence of left ventricular decompensation. However, many patients with aortic stenosis are comorbid with multifactorial dyspnoea, and it can be difficult to ascertain whether aortic valve intervention will improve symptoms and quality of life in these situations.^9^ Additionally, with the advent of transcatheter aortic valve implantation (TAVI), frail patients in whom surgical aortic valve replacement (SAVR) is deemed too high risk now have a less invasive intervention available,^10^ and the procedural risks and costs must be balanced against the potential prognostic and/or symptomatic benefits.

Balloon aortic valvuloplasty (BAV) was first described in the 1980s and was performed in patients with severe aortic stenosis who were unfit for SAVR.^11^ Initially, the procedure was associated with high complication rates and offered only temporary symptomatic benefit.^12–15^ In addition, long-term survival following BAV was poor, with no improvement in the natural history of the underlying disease.^14^ As such the procedure came out of favour somewhat and was largely reserved for palliation.^14–17^ More recently, improvement in procedural techniques, equipment and patient assessment together with the introduction of TAVI has led to a revival in the use of BAV.^18 19^ Current guidelines offer weak recommendations for BAV as a bridge to definitive therapy in haemodynamically unstable patients or patients who require urgent, major non-cardiac surgery or as a test for symptom improvement (therapeutic response) before proceeding to aortic valve replacement.^6–8^ Consequently, the contemporary role of BAV as a an emergent procedure, a diagnostic adjunct to assess for symptom improvement or palliative measure must be carefully considered on an individual patient basis within the context of each institution’s heart team.

In this retrospective study of a contemporary cohort in the TAVI era, we characterise the use of BAV and determine its associations with clinical outcomes in a high-volume TAVI centre.

## Methods

### Study population

We reviewed demographic, clinical, echocardiographic and outcome data for all patients who underwent BAV for severe aortic stenosis between January 2010 and March 2018 at the Edinburgh Heart Centre, a tertiary cardiology centre in the Southeast of Scotland. TAVI was introduced in October 2012, and during the study period, the Edinburgh Heart Centre was the sole TAVI centre in Scotland, receiving all nationwide referrals. Decisions for TAVI or BAV as a bridging strategy to TAVI are made by the heart team. Patients who did not have a Community Health Index (CHI) number, assigned to all patients residing in Scotland were excluded from the current analysis.

### BAV procedure

All valvuloplasties were performed via a percutaneous transfemoral arterial approach. Ultrasound-guided access was not mandated over this time period. Several commercial valvuloplasty balloons were available (Valver-Balton, Poland; Cristal-Balt, France; NuCLEUS-NuMED, New York, USA; TRUE-Bard, Arizona, USA) with arterial sheath size varying between 8 and 12-French depending on balloon size. All procedures were undertaken under rapid ventricular pacing, with transfemoral venous access being standard for the apical placement of a 5 or 6-French temporary pacing wire. Therapeutic anticoagulation is standard, using an intravenous dose of 70–100 IU/kg unfractionated heparin. While during the initial study period the procedure was mostly performed for symptom palliation or as a bridge to SAVR in a small proportion of patients, following introduction of TAVI the indications for BAV have expanded in line with the international guidelines on the management of valvular heart disease. BAV was, therefore, also considered as a bridge to percutaneous aortic valve replacement or as a test for symptom improvement to aid definitive management decisions. Absolute contraindications for BAV included metallic aortic valve prostheses, active aortic valve endocarditis and concomitant severe aortic regurgitation.

### Data collection

Patient demographics, clinical history including prior comorbidities were collected from a standardised electronic patient record (TrakCare; InterSystems Corporation, Cambridge, Massachusetts, USA). For the purpose of this analysis, LVEF was defined as normal (EF >50%), moderate (EF 30%–49%) and poor (EF <30%). The procedural priority was defined as elective, urgent (unplanned inpatient procedure) and emergency (unplanned inpatient procedure in a critically unwell patient (cardiogenic shock, mechanical or inotropic support). Available echocardiographic information in terms of aortic stenosis parameters and left ventricular systolic function preintervention and postintervention were obtained from integrated clinical records. Clinical outcomes were determined from individual patient data linkage from the National Scottish Morbidity Record (SMR01), Information Services Division, Scotland with relevant International Classification of Diseases (ICD-10) and Office of Population Censuses and Surveys Classification of Interventions and Procedures (OPCS) codes (Online supplemental appendix 1). This is a national registry which captures all deaths and inpatient hospital admissions. Qualitative data on symptomatic status pre-BAV and post-BAV were recorded from clinical records where available.

10.1136/openhrt-2020-001330.supp1 Supplementary data

### Clinical outcomes

The primary endpoint was all-cause mortality. Thirty-day and 12 months all-cause mortality were also reported. Secondary endpoints included 30 days myocardial infarction, stroke, heart failure (new), acute renal failure, bleeding and symptomatic improvement. Clinical outcomes were defined by relevant diagnostic ICD-10 and OPCS codes (Online supplemental appendix 1). Rates and type of repeat intervention after the index BAV procedure were also collected.

### Statistical analysis

Baseline characteristics are presented as frequencies and percentages for categorical data and as median values with IQR for continuous data. These are presented for the overall study population and between-group comparisons performed using a two-sample t-test. Cumulative mortality was assessed using Kaplan-Meier curves and the log-rank test stratified by repeat intervention and index BAV procedure priority. Cox regression models for all-cause mortality were constructed to identify independent predictors of survival, adjusted for age, sex, hypertension, ischaemic heart disease, atrial fibrillation, chronic kidney disease, diabetes, pulmonary disease, cerebrovascular disease, priority of BAV procedure and LVEF category, which were identified a priori as clinically relevant. Adjusted time-to-event curves stratified by predictors of mortality were constructed for the multivariable Cox regression model. Qualitative symptomatic benefit is reported as a dichotomous variable. Univariable and multivariable logistic regression models, adjusted for prespecified clinical factors relevant to symptomatic status (age, sex, ischaemic heart disease, pulmonary disease, LVEF, procedure priority, baseline mean gradient and change in mean gradient) were constructed to identify independent factors associated with symptomatic improvement. A two-sided p<0.05 was taken to be significant. Analyses were performed using R V.3.5.0 (R Foundation for Statistical Computing, Vienna, Austria).

## Results

**Figure 1:**
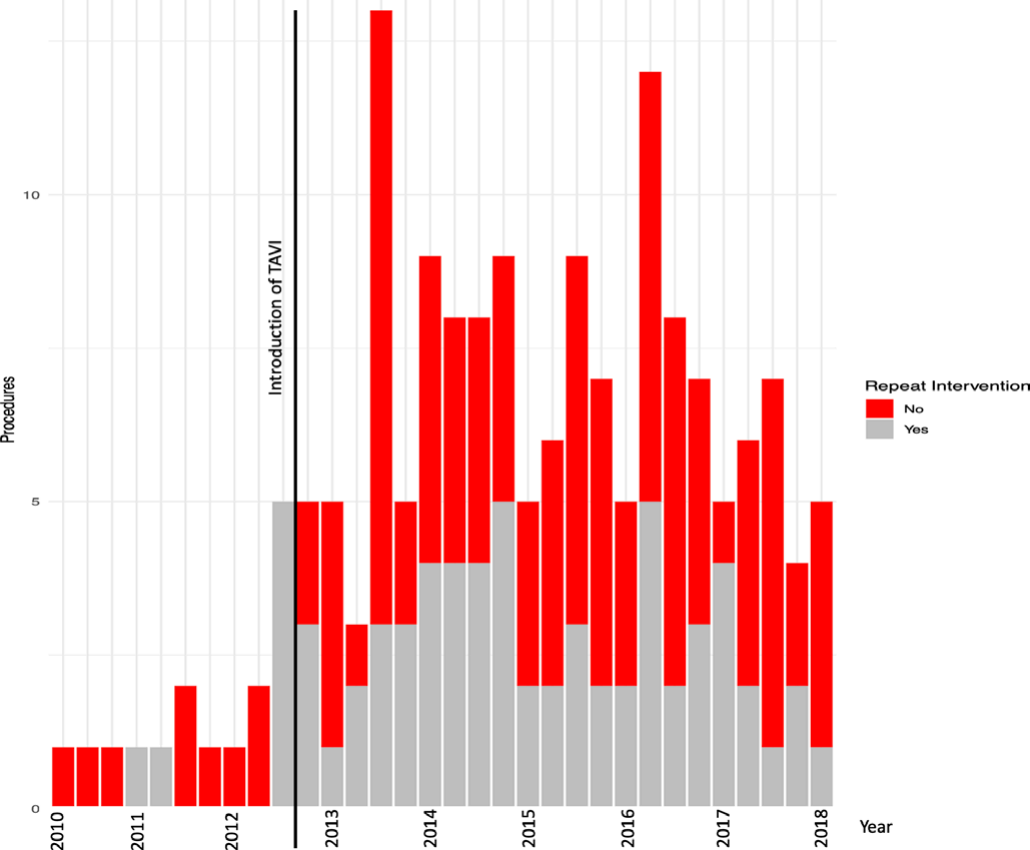
Quarterly rates of BAV and subsequent repeat intervention during the study period of January 2010 to March 2018. (TAVI was introduced in the Edinburgh Heart Centre in the last quarter of 2012.) Online supplemental table 3 outlines the number of procedures per year. BAV, balloon aortic valvuloplasty; TAVI, transcatheter aortic valve implantation.

Between January 2010 and March 2018, a total of 168 patients underwent BAV. One patient without a personal identifier (CHI number) was excluded, leaving 167 patients in the final cohort with a median follow-up time of 11.1 (IQR 3.5–27.5) months from the index procedure. Of these, 67 patients underwent repeat aortic valve intervention: 42 TAVI, 16 SAVR and 9 repeat BAV. There was a temporal change in use of BAV, with more annual procedures from 2013 onwards. In addition, the number of repeat interventions following the index BAV also increased over time with a large proportion of patients undergoing a follow-up procedure (figure 1, Online supplemental table 1).

### Population characteristics

The BAV cohort had a balanced gender distribution. The cohort was elderly with a median age of 80 (IQR 73–86) years and a high burden of medical comorbidities (table 1). The index procedure was elective in 79 (47%), urgent in 72 (43%) and emergent in 16 (10%) patients. Patients undergoing an emergency BAV were younger and had an increased prevalence of both impaired renal function and EF <30%. The indications for emergency BAV were cardiogenic shock and/or multiorgan failure, while all urgent procedures were performed for decompensated heart failure with worsening symptoms despite appropriate medical therapy.

**Table 1:** Baseline characteristics according to index BAV priority

	Overall	Elective	Urgent	Emergency	P value
N	167	79	72	16	
Age (IQR)	80.0 (73.0 to 86.0)	81.0 (73.0 to 87.0)	81.0 (74.0 to 87.0)	74.5 (69.5 to 77.2)	0.011
Male (%)	87 (52.1)	40 (50.6)	38 (52.8)	9 (56.2)	0.909
Hypertension (%)	100 (59.9)	48 (60.8)	42 (58.3)	10 (62.5)	0.931
Ischaemic heart disease (%)	67 (40.1)	28 (35.4)	31 (43.1)	8 (50.0)	0.443
Previous MI (%)	38 (22.8)	15 (19.0)	19 (26.4)	4 (25.0)	0.542
Previous PCI (%)	22 (13.2)	10 (12.7)	9 (12.5)	3 (18.8)	0.786
Previous CABG (%)	31 (18.6)	18 (22.8)	9 (12.5)	4 (25.0)	0.210
Cerebrovascular disease (%)	20 (12.0)	10 (12.7)	8 (11.1)	2 (12.5)	0.956
eGFR (ml/min/1.73m2)					<0.001
>60 (%)	88 (52.7)	53 (67.1)	30 (41.7)	5 (31.2)	
30–59 (%)	55 (32.9)	22 (27.8)	29 (40.3)	4 (25.0)	
<30 (%)	20 (12.0)	4 (5.1)	10 (13.9)	6 (37.5)	
Haemodialysis	4 (2.4)	0 (0.0)	3 (4.2)	1 (6.2)	
Diabetes (%)	43 (25.7)	17 (21.5)	24 (33.3)	2 (12.5)	0.112
Pulmonary disease (%)	47 (28.1)	24 (30.4)	18 (25.0)	5 (31.2)	0.732
Peripheral vascular disease (%)	22 (13.2)	13 (16.5)	9 (12.5)	0 (0.0)	0.202
Prior pacemaker (%)	9 (5.4)	2 (2.5)	7 (9.7)	0 (0.0)	0.089
LVEF (%)					<0.001
>50%	64 (38.8)	47 (60.3)	14 (19.7)	3 (18.8)	
**30%–50%**	47 (28.5)	15 (19.2)	30 (42.3)	2 (12.5)	
<30%	54 (32.7)	16 (20.5)	27 (38.0)	11 (68.8)	
Smoking history					0.529
No (%)	101 (68.2)	51 (69.9)	40 (63.5)	10 (83.3)	
Ex (%)	38 (25.7)	19 (26.0)	18 (28.6)	1 (8.3)	
Yes (%)	9 (6.1)	3 (4.1)	5 (7.9)	1 (8.3)	
Peak gradient (mm Hg) (IQR)	70.0 (57.0 to 80.0)	71.0 (56.2 to 84.0)	70.0 (58.0 to 79.0)	66.5 (54.8 to 73.8)	0.78
Mean gradient (mm Hg) (IQR)	42.0 (30.0 to 50.0)	42.5 (32.8 to 52.0)	43.5 (30.0 to 49.2)	38.0 (31.0 to 47.0)	0.655
NYHA class *					0.001
I (%)	5 (3.7)	2 (2.5)	1 (1.7)	2 (20)	
II (%)	41 (30.4)	29 (36.7)	9 (15.5)	3 (30)	
III (%)	68 (50.3)	31 (39.2)	34 (58.6)	3 (30)	
IV (%)	21 (15.5)	5 (6.3)	14 (24.1)	2 (20)	
Improved symptoms (%)†	75 (62.5)	43 (70.5)	25 (52.1)	7 (63.6)	0.143
Repeat intervention					<0.001
None	100 (59.9)	45 (57.0)	48 (66.7)	7 (43.8)	
BAV (%)	9 (5.4)	3 (3.8)	5 (6.9)	1 (6.2)	
Surgical AVR (%)	16 (9.6)	4 (5.1)	4 (5.6)	8 (50.0)	
TAVI (%)	42 (25.1)	27 (34.6)	15 (20.8)	0 (0.0)	

*135 patients in total with available NYHA class, 67 in the elective group, 58 in the urgent group and 10 in the emergency group.

†120 patients in total with available symptomatic status post index BAV, 75 of these had symptom improvement, 43 in the elective group, 25 in the urgent group and 7 in the emergency group.

AVR, aortic valve replacement; BAV, balloon aortic valvuloplasty; CABG, coronary artery bypass surgery; eGFR, estimated glomerular filtration rate; LVEF, left ventricular ejection fraction; MI, myocardial infarction; NYHA, New York Heart Association; PCI, percutaneous coronary intervention; TAVI, transcatheter aortic valve implantation.

### Haemodynamics and symptoms

Baseline LVEF was <50% in 101 patients (60.4%) of the study cohort. Preprocedure echocardiographic mean gradient was available in 121 (72%) patients. The median time from preprocedure echocardiogram to the index BAV was 42 (IQR 8–130) days and the median pre-procedure mean gradient was 42 (IQR 30–50) mm Hg. Postprocedure echocardiographic mean gradient was available in 108 (65%). The median time to postprocedure echocardiogram was 8 (IQR 1–58) days and the median postprocedure mean gradient was 32 (IQR 25–42) mm Hg. The median change in mean gradient was 8 (IQR 1–16) mm Hg.

New York Heart Association class prior to the index BAV was available in 135 (80.8%) patients, most of whom were class II and III (table 1). A larger proportion of patients requiring urgent or emergent intervention were class III or IV compared with those undergoing elective. Symptomatic status at 3 months follow-up after the index BAV was available in 120 (72%) patients, 61 (51%) of whom subsequently underwent repeat intervention. A total of 75 (62.5%) patients described symptomatic improvement. A greater proportion of patients with symptomatic improvement went on to have a repeat intervention (46 of 75, 61%) compared with those who did not have symptomatic improvement (13 of 45, 29%). In a multivariable logistic regression model, change in mean gradient was not associated with symptom improvement (OR (OR) 1.06, 95% CI 0.98 to 1.16), nor were other independent predictors identified.

### Clinical outcomes

**Figure 2:**
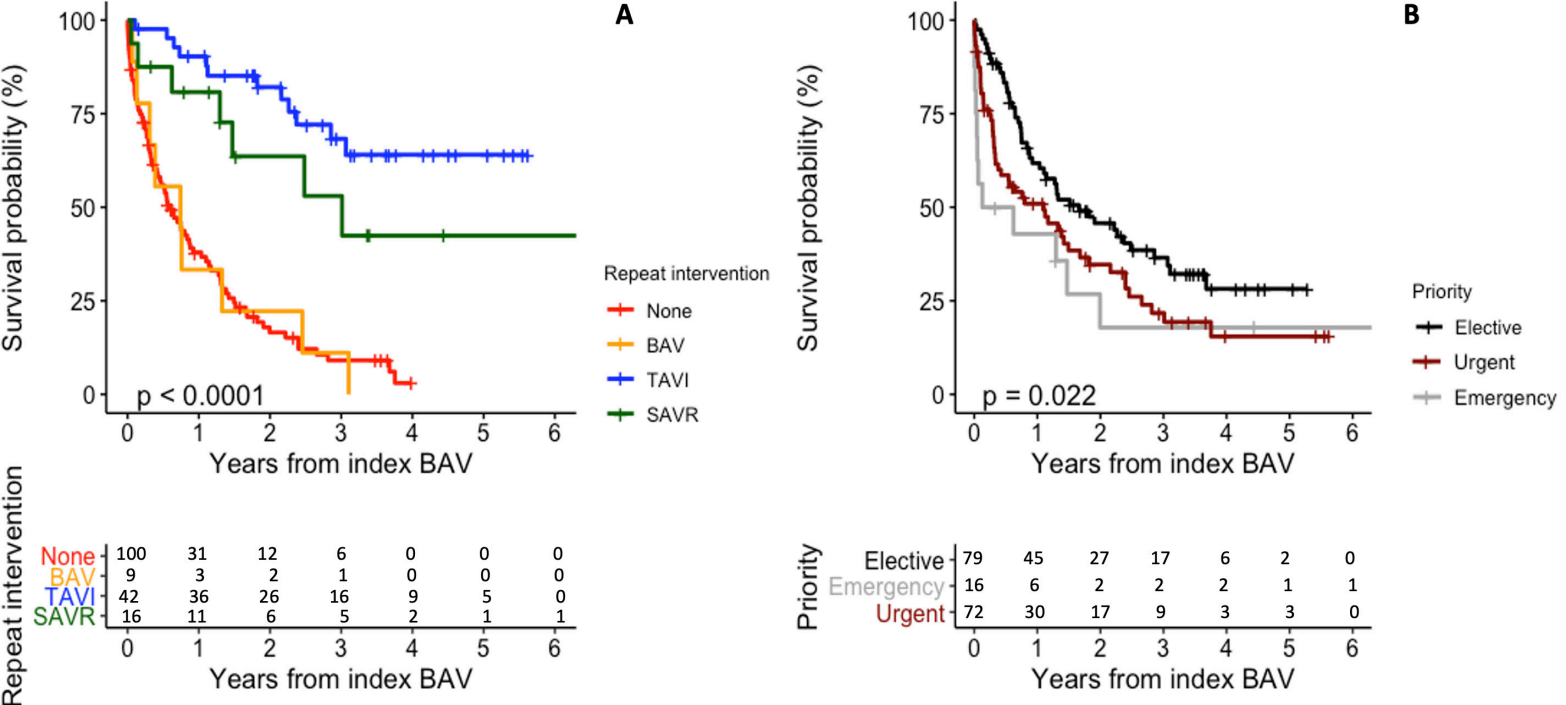
Panel plot showing Kaplan-Meier survival curves and corresponding number at risk tables stratified by (A) repeat intervention and (B) index BAV procedure priority. Groups are compared using the log-rank test. BAV, balloon aortic valvuloplasty; SAVR, surgical aortic valve replacement; TAVI, transcatheter aortic valve implantation.

All-cause mortality for the study duration was 67% with a median survival of 212 (IQR 54–490) days. Thirty-day and 12-month mortality were 11% and 43%, respectively. In unadjusted analysis, male gender, procedural priority (both urgent and emergency) and reduced LVEF were associated with all-cause mortality (Online supplemental table 2). There was a small difference in adjusted mortality when stratified by index BAV procedure priority, with patients in the elective group showing minor improvement in long-term survival (figure 2B). After adjustment, reduced EF (EF 30%–50%: HR 1.76, 95% CI 1.05 to 2.94, p=0.031; EF <30%: HR 1.90, 95% CI 1.12 to 3.20, p=0.017) remained a strong independent predictor of mortality (table 2) with improved survival probability in patients with good EF when compared with those with reduced EF (Online supplemental figure 1).

**Table 2:** Multivariable Cox model for all-cause mortality

	HR	P value
Age	0.85 (0.67–1.11)	0.231
Sex (male)	1.37 (0.91–2.06)	0.128
Hypertension	1.23 (0.81–1.85)	0.330
Ischaemic heart disease	1.18 (0.79–1.77)	0.425
Atrial fibrillation	1.30 (0.84–1.98)	0.240
Chronic kidney disease	1.43 (0.82–2.48)	0.209
Diabetes	0.86 (0.53–1.40)	0.545
Pulmonary disease	1.25 (0.79–1.96)	0.336
Cerebrovascular disease	1.32 (0.74–2.37)	0.346
Priority—elective	Reference	
Urgent	1.12 (0.71–1.74)	0.631
Emergency	1.59 (0.79–3.20)	0.197
LVEF category—good >50%	Reference	
Moderate 30%–50%	1.76 (1.05–2.94)	0.031
Poor <30%	1.90 (1.12–3.20)	0.017

LVEF, left ventricular ejection fraction).

There were two procedural deaths recorded. Both patients had multiorgan failure prior to the procedure and underwent emergency BAV. A bleeding event occurred in one patient (0.6%), while two patients (1.2%) had a stroke (Online supplemental table 3).

### Repeat intervention

In those who underwent subsequent intervention, the median time from index to repeat procedure was 161 (IQR 61–253) days. Patients undergoing TAVI were older with more peripheral vascular disease, while patients undergoing SAVR had higher aortic valve gradients (Online supplemental table 4). Of the nine patients who underwent repeat BAV, three had the second procedure prior to the introduction of TAVI and were not suitable candidates for SAVR. The remaining six underwent repeat BAV following TAVI MDT discussion, based on symptomatic response following the initial intervention but limited life expectancy due to other significant comorbidities.

Patients undergoing an elective procedure were more likely to have repeat intervention in the form of TAVI, while half of those who had an emergency procedure underwent SAVR subsequently—there were no TAVIs performed in this subgroup. When stratified by repeat intervention, there was a marked difference in mortality between treatment strategies (figure 2A). Mortality at 30 days and 12 months was highest in the nine patients who underwent repeat BAV, followed by those who did not undergo repeat intervention (Online supplemental table 5, figure 2). The median survival following repeat BAV was 37 (IQR 26–178) days; six of nine patients died within 2 months.

## Discussion

In this retrospective cohort study of consecutive patients undergoing BAV for severe aortic stenosis, we have demonstrated that the use of BAV increased substantially following the introduction of TAVI. We show that BAV can be performed with a low procedural risk of death or stroke, improves symptoms in some patients, and when used as a bridge to definitive aortic valve intervention can offer good outcomes in the current era. In the small proportion of patients who underwent a repeat BAV, the mortality rate was very high.

Contemporary registry data have shown that the use of BAV has increased in the past decade since a considerable number of high-risk patients with severe aortic stenosis are considered for percutaneous valve intervention procedures.^20–22^ Concomitantly, an improvement in procedural technique has also contributed to this change.^13^ We have confirmed these prior observations and additionally found that the rate of BAV procedures increased substantially on the introduction of TAVI, with a large proportion of patients undergoing a follow-up intervention — predominantly TAVI. The demographic and clinical characteristics of patients undergoing BAV observed in our retrospective analysis are similar to those found in other cohort studies.^21–23^ Patients undergoing BAV are elderly with a balanced gender distribution and have a range of comorbidities include hypertension, ischaemic heart disease, renal disease, pulmonary disease and diabetes. These characteristics are similar to those of patients undergoing TAVI in current clinical practice.

Kapadia *et al*^24^ reported on the impact of BAV in a subgroup analysis of the seminal Placement of AoRtic TraNscathetER Valves trial. Survival at 1 year in patients undergoing BAV was poor and was similar in those who had standard medical care. However, BAV did improve 3-month survival and quality of life up to 6 months. Our findings are consistent with these data, demonstrating poor survival without definitive intervention and no change in mortality with BAV, but a short-term improvement in symptoms with acceptable procedural safety. As consecutive patients were analysed, we were able to show, as expected, a marked survival benefit in patients undergoing definitive intervention — clearly a function of patient selection.

The use of repeat BAV in patients with symptomatic severe aortic stenosis who developed restenosis following the index procedure has been a palliative strategy in previous years. Observational data have reported repeat BAV to be associated with improved symptom-free survival to 3 years in patients who were unfit for surgical intervention with acceptable procedural complication rates.^25 26^ However, these findings were seen predominantly in the pre-TAVI era, where surgical intervention remained the only definitive procedure. Thus, patients selected for repeat BAV in the current era are likely to be frailer and more comorbid. Congruent with this, we observed that the few patients who underwent repeat BAV had a very high mortality rate and a median survival from repeat BAV of only 1 month.

The importance of patient assessment and understanding the rationale for BAV — either for emergent afterload relief in the context of haemodynamic compromise, diagnostic purposes or palliation—is clear. We found reduced LVEF to be a strong and independent predictor of mortality, as expected, but not symptom improvement. This may speak to the difficulties in assessing the relationship between symptoms and functional haemodynamics in low flow states — a complex clinical milieu. Emergency index BAV was an unadjusted predictor of mortality, but this association was not present in multivariable modelling. This likely represents selection bias—that is, it is probable that only those judged most likely to achieve a favourable clinical outcome were selected for emergency BAV. Unstable, acutely decompensated aortic stenosis is difficult to manage, with questions open as to what interventions should be considered in the context of each patient and each centre’s logistic capabilities. Prior observational data have not demonstrated a benefit to emergent TAVI over emergent BAV, but staged TAVI following emergent BAV was associated with a higher than expected procedural and short-term mortality.^27 28^ This is an uncertain clinical area with a need for robust data.

Although other clinical factors may be expected to contribute to outcomes, we found only small differences between groups. There are multiple assessments of frailty and physiological reserve that are validated and have prognostic relevance^29^ but are not captured in the collection of routine data such as comorbid conditions and cardiac haemodynamics. Indeed, although current guidelines support the use BAV as a bridge to definitive intervention,^6–8^ universally adopted criteria on how to identify these patients do not exist. These evaluations must be individualised, using the expertise of the local multidisciplinary team. In our cohort, we demonstrated favourable outcomes in patients who underwent a definitive aortic valve intervention following BAV, providing useful real-world data on contemporary use of BAV in the TAVI era and support the use of BAV as a therapeutic trial or bridge to definitive intervention in carefully selected patients.

The strengths of this study are the evaluation of a consecutive cohort of patients undergoing BAV. The use of Scottish Morbidity Records (SMR01) allows robust linkage with clinical outcomes. The 8-year study period spans the pre-TAVI and post-TAVI era in our institution, and furthermore captures all subsequent TAVIs in these patients due to the nationwide referral system. However, there are several important limitations. This was a retrospective single-centre cohort study with the attendant issues that accompany this study design — in particular, unmeasured confounders and selection bias and our findings must be interpreted in this context. It is reassuring, however, that our findings are largely in keeping with the existing body of literature. We used data linkage to obtain accurate and comprehensive national outcome data. However, there was incomplete clinical or investigation-related data for some patients, including objective frailty assessment and detailed echocardiographic variables. Furthermore, although routine data linkage is very robust for mortality, morbidity was not adjudicated in this study, and may therefore be subject to inaccuracy, although our reported findings are largely congruent with other observational cohorts. Finally, symptomatic status was not able to be formally assessed retrospectively and was thus qualitative only; validated metrics of quality of life were not routinely performed before or after the procedure.

## Conclusion

In contemporary practice in the TAVI era, long-term outcomes in patients selected for an isolated BAV remain guarded. However, BAV may still play a useful role in improving symptoms and as a bridge to definitive intervention in carefully selected patients with symptomatic severe aortic stenosis.
