# Supplementary material for: Clinical outcomes following balloon aortic valvuloplasty
Source: Open Heart. 2020 Sep 8;7(2):e001330. doi: 10.1136/openhrt-2020-001330 (PMC7481086; doi:10.1136/openhrt-2020-001330)

Supplementary Material

Clinical outcomes following balloon aortic valvuloplasty

Anda Bularga MD[1], Rong Bing MBBS[1,2], Anoop S. V. Shah MD[1,2], Philip D. Adamson MBBS[2,3], Miles Behan MD[2], David E. Newby MD[1,2], Andrew D. Flapan MD[2], Neal Uren MD[2], Nick Cruden MD[2]

[1] BHF Centre for Cardiovascular Science, University of Edinburgh, Edinburgh, UK.

[2] Edinburgh Heart Centre, Royal Infirmary of Edinburgh, Edinburgh, UK

[3] Christchurch Heart Institute, University of Otago, New Zealand

All authors take responsibility for all aspects of the reliability and freedom from bias of the data presented and their discussed interpretation.

Appendix 1*:* ICD-10 codes for secondary outcomes at 30 days and 1 year

Myocardial Infarction: I210, I2100, I2101, I2109, I211, I2110, I2111, I2119, I212, I2120, I2121, I2129, I213, I2130, I2131, I2139, I214, I2140, I2141, I2149, I219, I2190, I2191, I2199, I220, I2200, I2201, I2209, I221, I2210, I2211, I2219, I228, I2280, I2281, I2289, I229, I2290, I2291, I2299

Stroke: I609, I602, I638 D, I614, I600, I631 D, I63, I631, I632, I607, I616, I633, I6390, I64X, I603, I632 D, I635, I608, I618, I634, I606, I639 D, I630, I64X D, I613, I612, I619, I620, I615, I610

Heart failure: I509, I500, I500 D, I501, I5009, I501 D, I426, I5010, I5090, I110, I5000, I5019, I5099, I5091, I5001, I5001D, I509 D, I5011, I5009D, I130, I132, I5000D, I5010D, I5091D, I5019D, I5099D, I1329, I5090D, I1109, I1101, I1100, I5011D, I1320, I1321, I1300, I1309

Acute Renal Failure: N17

Bleeding: H431, K920, K264, R042, K922, I601, I609, K921, I620, K625, K260, I629, K226, N938, S066, O041, I608, N939, T810, S0650, K250, I607, S065, I602, K290, I850, K266, S0660, K254, I603, H450 A, I604, I621, I606, K291, S0640, O722, O209, I230, I605, K262, R048, K284, K276, S0641, K256, H356, K274, O071, S064, O208, O036, S0651, O721, K252, R041, K286, S0661, K270, I312, N837, O081, O031, K280, O046, O720, O469, R049, O902, I690, K272, I609 D, O076, I692, P261, O061, O051, K282, O066, O460, P269, O717, I607D

Supplementary table 1: Number of index BAV and repeat interventions (January 2010 to March 2018)

Year	Number of index BAV procedures per year	Number of patients undergoing repeat intervention
2010	3	0
2011	5	2
2012	13	8
2013	26	9
2014	34	17
2015	27	9
2016	32	12
2017	22	9
2018	5	1

BAV=balloon aortic valvuloplasty

**Supplementary table 2 – Univariable Cox model for all-cause mortality**		
	**Hazard ratio**	**p-value**
**Age**	0.92 (0.75-1.13)	0.404
**Sex (male)**	1.50 (1.03-2.19)	0.034
**Hypertension**	1.10 (0.75-1.61)	0.617
**Ischaemic heart disease**	1.40 (0.96-2.03)	0.083
**Atrial fibrillation**	1.10 (0.75-1.60)	0.628
**Chronic Kidney Disease**	1.46 (0.91-2.37)	0.12
**Diabetes**	1.07 (0.70-1.63)	0.762
**Pulmonary disease**	1.01 (0.67-1.52)	0.959
**Cerebrovascular disease**	1.32 (0.76-2.27)	0.323
**Priority - Elective**	Reference	
**Urgent**	1.55 (1.04-2.30)	0.03
**Emergency**	2.08 (1.10-3.92)	0.024
**LVEF category >50%**	Reference	
**30-50%**	1.82 (1.14-2.89)	0.012
**<30%**	2.06 (1.31-3.25)	0.002
(LVEF=left ventricular ejection fraction)		

**Supplementary table 3 – Thirty-day morbidity post index BAV**	
	**Events**
**MI (%)**	8 (4.8)
**Stroke (%)**	2 (1.2)
**Heart Failure (%)**	9 (5.4)
**Acute Renal Failure (%)**	14 (8.4)
**Bleeding (%)**	1 (0.6)
**All-cause mortality (%)**	18 (10.8)
(N=number, MI=myocardial infarction, ARF=acute renal failure)	

Supplementary table 4: Baseline characteristics according to repeat intervention type					
	**Overall**	**BAV**	**TAVI**	**SAVR**	**p-value**
**N**	167	9	42	16	
**Age [IQR]**	80.0 [73.0, 86.0]	74.0 [65.0, 78.0]	84.0 [73.2, 89.5]	74.0 [71.0, 78.2]	0.001
**Male (%)**	87 (52.1)	5 (55.6)	21 (50.0)	8 (50.0)	0.981
**Hypertension (%)**	100 (59.9)	6 (66.7)	23 (54.8)	9 (56.2)	0.824
**Ischaemic heart disease (%)**	67 (40.1)	5 (55.6)	17 (40.5)	6 (37.5)	0.803
**Previous MI (%)**	38 (22.8)	2 (22.2)	8 (19.0)	2 (12.5)	0.596
**Previous PCI (%)**	22 (13.2)	2 (22.2)	4 (9.5)	1 (6.2)	0.553
**Previous CABG (%)**	31 (18.6)	3 (33.3)	12 (28.6)	2 (12.5)	0.119
**Cerebrovascular disease (%)**	20 (12.0)	0 (0.0)	1 (2.4)	3 (18.8)	0.068
**eGFR (ml/min/1.73m2)**					0.315
**>60 (%)**	88 (52.7)	4 (44.4)	27 (64.3)	7 (43.8)	
**30-59 (%)**	55 (32.9)	4 (44.4)	14 (33.3)	4 (25.0)	
**<30 (%)**	20 (12.0)	1 (11.1)	1 (2.4)	4 (25.0)	
**Haemodialysis (%)**	4 (2.4)	0 (0.0)	0 (0.0)	1 (6.2)	
**Diabetes (%)**	43 (25.7)	3 (33.3)	10 (23.8)	2 (12.5)	0.554
**Pulmonary disease (%)**	47 (28.1)	2 (22.2)	14 (33.3)	4 (25.0)	0.835
**Peripheral vascular disease (%)**	22 (13.2)	0 (0.0)	10 (23.8)	0 (0.0)	0.045
**Pacemaker (%)**	9 (5.4)	0 (0.0)	1 (2.4)	0 (0.0)	0.320
**Smoking history**					0.728
**No (%)**	101 (68.2)	6 (66.7)	27 (69.2)	12 (85.7)	
**Ex (%)**	38 (25.7)	3 (33.3)	9 (23.1)	2 (14.3)	

**Yes (%)**	9 (6.1)	0 (0.0)	3 (7.7)	0 (0.0)	
**Priority of index BAV**					<0.001
**Elective (%)**	79 (47)	3 (33.3)	27 (64.3)	4 (25.0)	
**Urgent (%)**	72 (43.4)	5 (55.6)	15 (35.7)	4 (25.0)	
**Emergency (%)**	16 (9.6)	1 (11.1)	0 (0.0)	8 (50.0)	
**LVEF**					0.394
**>50%**	64 (38.8)	4 (44.4)	21 (51.2)	7 (43.8)	
**30-50%**	47 (28.5)	2 (22.2)	12 (29.3)	4 (25.0)	
**<30%**	54 (32.7)	3 (33.3)	8 (19.5)	5 (31.2)	
**Peak gradient (mmHg) [IQR]**	70.0 [57.0, 80.0]	68.0 [55.0, 76.5]	64.0 [53.5, 78.5]	75.0 [64.5, 90.0]	0.065
**Mean gradient (mmHg) [IQR]**	42.0 [30.0, 50.0]	40.0 [30.0, 48.0]	36.0 [29.0, 52.0]	49.0 [42.5, 55.0]	0.005
(IQR=interquartile range, MI=myocardial infarction, PCI=percutaneous coronary intervention, CABG=coronary artery bypass surgery, eGFR=estimated glomerular filtration rate, LVEF=left ventricular ejection fraction, NYHA=New York Heart Association)					

Supplementary table 5 – All-cause mortality stratified by repeat intervention

	Overall	None	Repeat BAV	TAVI	SAVR
**N**	167	100	9	42	16
**Mortality at 30 days (%)**	18 (11%)	16 (16%)	3 (33%)	3 (7%)	1 (6%)
**Mortality at 12 months (%)**	72 (43%)	60 (60%)	8 (89%)	6 (14%)	3 (19%)

(N=number, BAV=balloon aortic valvuloplasty, TAVI= transcatheter aortic valve implantation, SAVR=surgical aortic valve replacement)

Supplementary figure 1: Multivariable Cox proportional hazard survival stratified by baseline ejection fraction.

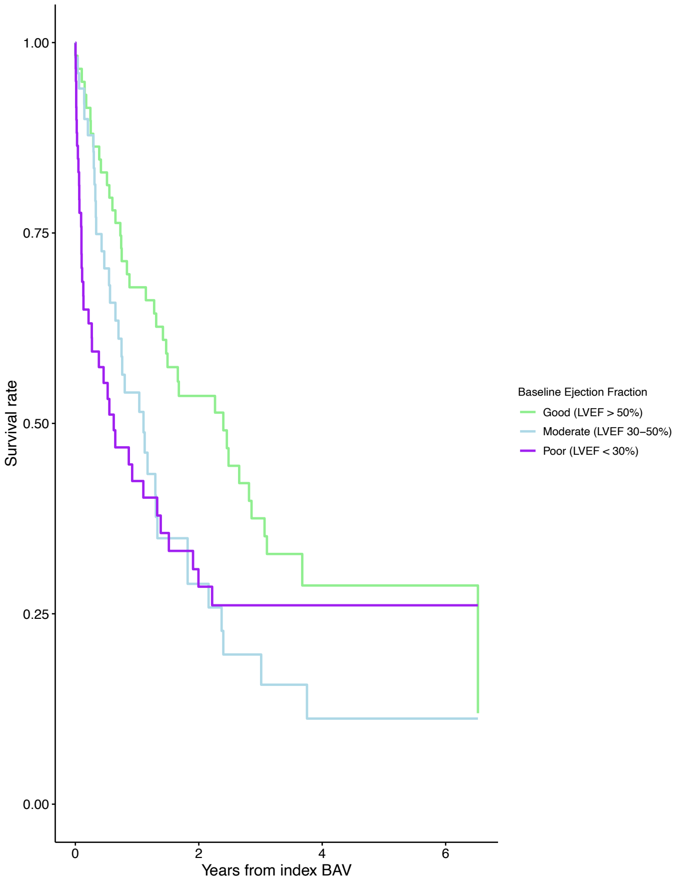

Supplement: Supplementary data [file openhrt-2020-001330supp001.pdf]
